# Evaluation of the Seismic Behavior of Carbon-Grid-Reinforced Walls with Varying Anchorage and Axial Load Ratios

**DOI:** 10.3390/polym18010144

**Published:** 2026-01-05

**Authors:** Kyung-Min Kim, Sung-Woo Park, Bhum-Keun Song, Kyung-Jae Min, Seon-Hee Yoon

**Affiliations:** 1 Seismic Safety Center, Korea Conformity Laboratories (KCL), Suwon 16229, Republic of Korea; sungwoo@kcl.re.kr; 2 Korea Carbon Industry Promotion Agency, Jeonju 54853, Republic of Korea; bksong@kcarbon.or.kr (B.-K.S.); bluemy@kcarbon.or.kr (K.-J.M.); 3 High In Tech Co., Ltd., Ulsan 44785, Republic of Korea; w470470@nate.com

**Keywords:** reinforced concrete, fiber-reinforced polymer, grid-type carbon-fiber-reinforced polymer reinforcement, carbon-grid-reinforced concrete, seismic behavior

## Abstract

Fiber-reinforced polymers (FRPs) are being increasingly used to replace rebars as reinforcements for concrete. This study evaluated the seismic behavior of concrete walls reinforced with grid-type carbon FRP (CFRP; carbon grid) through quasi-static cyclic tests and compared the results with that of the reinforced concrete (RC) wall. The experimental variables were the ratio of the carbon-grid anchorage length in the foundation to the wall length and the axial force ratio. Based on the results of the quasi-static cyclic tests, the ratio of the equivalent stiffness at the crushing of the compression-edge cover concrete to the initial stiffness of the carbon-grid-reinforced concrete specimens was 0.14 on average. This indicates that the specimens reached their maximum load due to the crushing of the compression-edge cover concrete after a significant reduction in stiffness due to cracking. The skeleton curve for the carbon-grid-reinforced concrete specimens was found to be bilinear, with reduced stiffness due to cracking and failure due to the crushing of the compression-edge cover concrete, making it definable and predictable. Additionally, in specimens with a high axial force or small ratio of the anchorage length in the foundation to the wall length, some of the longitudinal CFRP strands fractured at the same time as they reached the failure load. Moreover, the load at the crushing of the compression-edge cover concrete of the carbon-grid-reinforced concrete specimen increased by 1.10 times with the increase in the axial force ratio and decreased by 0.96 times with the decrease in the ratio of the anchorage length in the foundation to the wall length. It was found to be 0.73–0.80 times the flexural strength based on the assumption of plane sections remaining plane. In comparison with RC specimen, the cumulative absorbed energy of the carbon-grid-reinforced concrete specimen began to decrease after a story drift ratio of 1%, and the cumulative absorbed energy up to the target story drift ratio of 3.0% was found to be 0.60–0.62 times that of the RC specimen.

## 1. Introduction

Reinforced concrete (RC) structures can experience damage such as superficial cracking when exposed to marine environments, allowing chloride ions to penetrate inside the members. This can lead to rebar corrosion, concrete damage, and ultimately, a rapid decline in the durability of the members and the structure [1,2,3]. Rebar corrosion can also affect the fatigue performance of RC structures, such as bridges [4]. As an alternative to prevent the deterioration of the durability of RC structures owing to salt damage, the application of fiber-reinforced polymer (FRPs) as concrete reinforcement instead of the corrosion-prone rebar is being researched [5,6,7]. FRP reinforcement has primarily been applied to bridges and other structures susceptible to salt damage under exposure to marine environments. The applications of FRPs are rapidly expanding owing to its excellent mechanical properties, such as light weight and high strength. Recently, it has been used in the construction of new structures, such as building slabs and walls, as well as in the repair and reinforcement of existing RC structures.

The FRP reinforcement currently being developed and used is mostly a bar-type with a circular cross section, with ribs formed on it [8]. The main constituent materials are glass fiber as the reinforcement and thermosetting resin as the matrix. Carbon fiber exhibits characteristics such as a light weight, high tensile strength and modulus of elasticity, low density, and excellent chemical resistance [9]. To enhance the practicality and applicability of the FRP reinforcement, research is being conducted on the applicability of carbon fiber as a reinforcing material [10,11,12,13], and also on developing thermoplastic resin-based FRP reinforcement to ensure real-time bendability [14,15].

The tensile performance of textiles used as reinforcement in textile-reinforced concrete (TRC) is reported to be improved by impregnating them with a thermosetting resin [16,17,18]. Thus, textiles impregnated with thermosetting resins can be classified as a type of FRP reinforcement. Unlike the bar-type FRP reinforcement, which has a cross-sectional shape similar to that of a rebar, this grid-type FRP reinforcement is characterized by thin, plate-like, or elliptical FRP strands arranged at regular intervals in both the warp and weft directions, with the strands in both directions integrated at the intersections to form a grid [13,14,15,16,17,18,19,20,21,22,23].

Grid-type FRP reinforcement uses thin FRP strands, allowing it to reduce the height of structural members when compared with that in the case of bar-type FRP reinforcement. Additionally, because the longitudinal and transverse reinforcements do not need to be assembled onsite, it offers construction advantages not only for new buildings but also for the repair and reinforcement of existing structures. Moreover, grid-type FRP reinforcement has been shown to improve the durability of concrete structures in marine environments, with grid-type FRP-reinforced ultra-high-performance concrete (UHPC) plates exhibiting the smallest reduction in tensile strength [24]. Leveraging the superior corrosion resistance and mechanical properties of grid-type FRP, Fan et al. [25] performed fatigue tests on UHPC tubular beams, employing grid-type carbon FRP (CFRP) as transverse reinforcement and bar-type GFRP as longitudinal reinforcement, and derived the fatigue endurance limit for fatigue-critical applications. However, the spacing between the FRP strands in the warp and weft directions in grid-type FRP reinforcement is narrower than the spacing in rebar and bar-type FRP reinforcements, which use the typical coarse aggregates (of approximately 25 mm). In some cases, mortar is applied because it is difficult to use a coarse aggregate [26,27]. Furthermore, because FRP surfaces are generally smooth owing to the use of a resin [28], various methods have been developed to ensure their adhesion with cementitious composites. A representative method used for grid-type FRP reinforcement is coating the surface with silica sand [29]. Such methods are not required for bar-type FRP reinforcement, which has ribs on the surface, similar to a rebar.

The grid-type FRP reinforcement differs from the bar-type FRP reinforcement in other aspects such as the cross-sectional and surface shapes and internal spacing within the member [28,30]. Therefore, the behavior of concrete members reinforced with grid-type FRP reinforcement under external loads may differ from that of RC members. FRP reinforcement, particularly bar-type FRP reinforcement, is primarily used as the longitudinal reinforcement for horizontal members such as slabs and beams. ACI 440.1R-15 [4] provides design standards for the flexure and shear of concrete horizontal members reinforced with bar-type FRP reinforcement. For the flexural design of non-prismatic sections, strength reduction factors of 0.55 to 0.65 are applied depending on the reinforcement ratio to account for the uncertain strength characteristics of bar-type FRP reinforcement and to prevent brittle failure of the member. Deflection is calculated by applying the effective second moment of area, which considers the reduced tension stiffening effect when compared with that in RC members. According to the bending test results of concrete members reinforced with grid-type FRP reinforcement, the effective moment of inertia of the grid-type FRP reinforcement is smaller than that of bar-type FRP reinforcement, as mentioned in ACI 440.1R-15 [4], resulting in significantly larger deflections under the same load [31,32]. Furthermore, it was found that depending on the experimental conditions such as carbon-grid specifacation, specimen cross-sectional height, and the amount of reinforcement provided by the carbon grid, concrete specimens reinforced with grid-type FRP reinforcement generally exhibited higher flexural performance than that achieved by applying the strength reduction factors specified in ACI 440.1R-15 [31,32].

Some researchers [33,34,35,36,37,38,39,40,41,42,43,44] applied TRC to vertical members such as walls and columns to evaluate the changes in the structural performance owing to the repair and reinforcement of precast and existing members. Some studies [33,34,35] focused on precast members and developed a textile-reinforced cement sandwich wall by using thin panels reinforced with grid-type FRPs as the structural elements forming the outer skin. These studies aimed to reduce the thickness of precast concrete sandwich walls, which consist of a core insulation material and outer-skin structural elements, while experimentally evaluating its compressive behavior. Other studies [36,37,38,39,40,41,42,43,44] have also demonstrated the effectiveness of mortar- or engineered cementitious composite (ECC)-based techniques for the repair and strengthening of existing defective or damaged RC members. In particular, the confining effect on compressive strength achieved by applying basalt [36,37,38] or carbon [39] textile-reinforced ECC to the column surfaces has been extensively investigated. Liu et al. [40] reported that CFRP textile-reinforced ECC effectively improved the load capacity, secant stiffness and ultimate displacement of fire-damaged RC columns, while basalt fiber-reinforced ECC significantly enhanced the performance of corrosion-damaged columns [41]. In the case of mortar-based systems, TRC has been demonstrated to be effective for confinement [42], seismic retrofit [43], and enhancing the performance of corrosion-damaged columns [44].

Due to the brittle failure or potential buckling of FRP reinforcement under compression, both ACI 440.1R-15 [5] and CAN/CSA-S806-02 [45] generally recommend using FRP reinforcement as primary longitudinal reinforcement in horizontal members, such as beams and slabs. Moreover, for seismic design, CAN/CSA-S806-02 [45] permits FRP reinforcement as transverse reinforcement. FRP-reinforced concrete has recently been applied to structural walls, particularly in low-rise buildings where axial compression and seismic demands are relatively low [12]. A broader application of FRP reinforcement to structural walls requires a clear understanding of their behavior under axial loads, as well as their seismic behavior—including failure modes, deformation capacity, and energy absorption. However, conventional experimental studies on horizontal members, such as slabs and beams, remain limited [28,30,31,32,46,47,48], while experimental studies on vertical members, such as columns and walls, under loading conditions (i.e., seismic load) are particularly scarce—let alone novel studies employing machine learning approaches [49].

This study focused on adhesively bonded grid-type CFRP reinforcement (hereinafter referred to as the carbon grid, K-carbon, KC) and experimentally evaluated concrete walls reinforced with carbon grids, providing new insights into their failure modes, deformation capacity, and energy absorption, which are essential for seismic design.

Carbon grid KC consists of thin, flat CFRP strands manufactured with carbon fiber and epoxy through a pultrusion process. These strands are arranged in the warp and weft directions, and an adhesive is applied at the strand intersections to form the grid. Furthermore, the surface of the CFRP strands is treated with a peel ply to increase the surface area, thereby improving their adhesion with concrete.

In this study, carbon grid KC was used as the reinforcement for concrete walls, and quasi-static cyclic tests were conducted to investigate the seismic behavior. The main experimental variables were the ratio of the anchorage length of the carbon grid in the foundation to the wall length and the axial load ratio. Through quasi-static cyclic tests on RC wall specimens, the performance of the carbon grid KC as reinforcement for concrete was compared and evaluated against that of a rebar. The carbon-grid-reinforced concrete specimen exhibited a smaller extent of cover concrete spalling as flexural cracks progressed to flexural-shear cracks, along with greater deformation capacity than that of the RC specimen, establishing the suitability of carbon grid as a reinforcement material for concrete.

## 2. Materials and Methods

### 2.1. Experimental Design

#### 2.1.1. Material Properties

**Figure 1:**
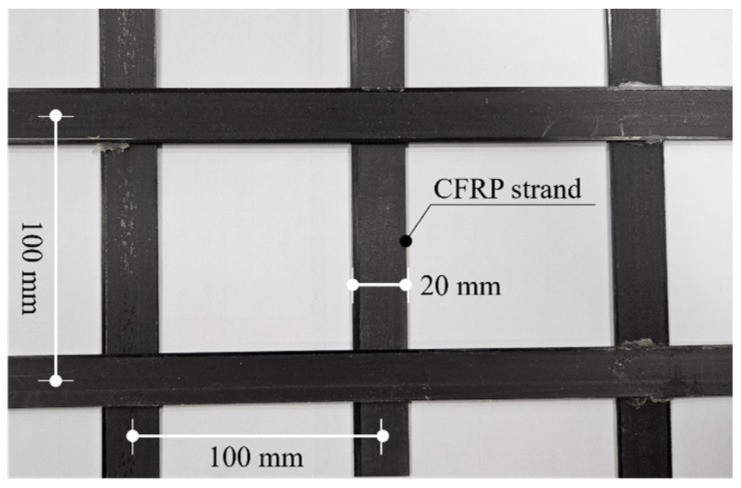
Carbon grid.

Figure 1 illustrates the carbon grid KC used in this study. KC has a carbon fiber content of 70 vol.% and is produced using automated equipment [31]. High-strength carbon fiber (Hyosung, Jeonju, Republic of Korea, H2550 12K) and a three-component low-viscosity liquid epoxy-based system (Huntsman, The Woodlands, TX, USA, ARALDITE^®^ CY 5192-1) were used. CFRP strands with a thin, flat plate-like shape of 20 × 1 mm (width × thickness) were fabricated by impregnating carbon fiber with epoxy and drawing them through a pultrusion process. Thereafter, by using automated production equipment, horizontal and vertical CFRP strands were bonded with a high-strength, fast-curing methyl methacrylate (MMA) adhesive (Bostik, Puteaux, France, SAF 30-15) with a lap shear strength of over 20 MPa to form a grid. The CFRP strands were first arranged horizontally at 100 mm intervals, and MMA adhesive was applied at the contact points with the vertical CFRP strands. Then, the vertical CFRP strands were placed at 100 mm intervals, and the contact points between the horizontal and vertical CFRP strands were compressed to fabricate the specimen. The surface of each CFRP strand was embossed using a peel ply treatment [50] to increase the specific surface area and improve the bond performance with concrete.

**Figure 2:**
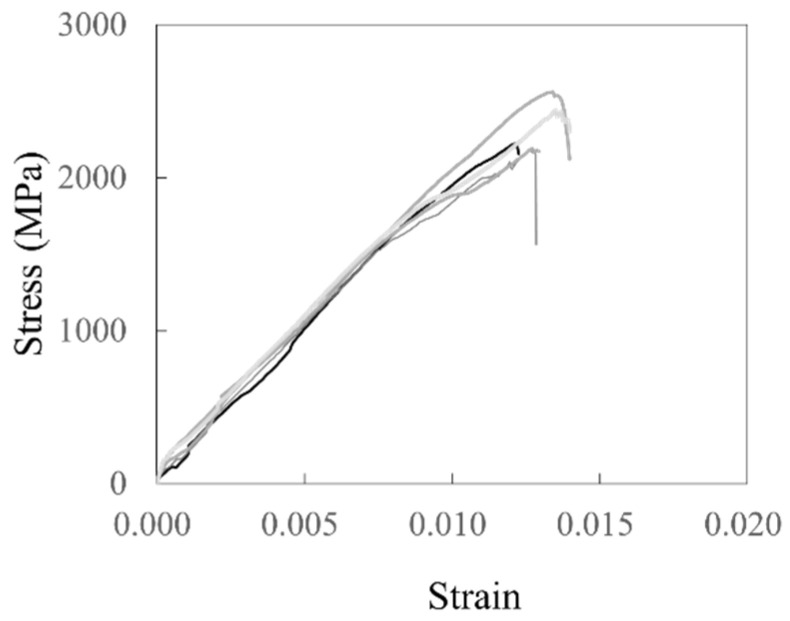
Material test results for CFRP strand samples.

Table 1 summarizes the characteristics of carbon grid KC and the rebar, as well as the tensile test results of the CFRP strands with carbon grid KC and the rebar. The tensile strength and tensile modulus of the CFRP strands were obtained by applying a load at a speed of 5 mm/min using a 1000 kN universal testing machine to tensile specimens fabricated by reinforcing both ends of the CFRP strands with steel plates and filling the space between the plates with epoxy, as recommended in ASTM D7205 [51] and CAN/CSA-S806-02 [45]. The CFRP strands failed when the load rapidly decreased at the same time as they reached their tensile strength, as depicted in Figure 2. In addition, the rebar was subjected to tensile testing according to ISO 6935 [52], and the yield strength, tensile strength, and tensile modulus of elasticity were determined. In the carbon grid, the tensile strength of the CFRP strands that constitute the KC is approximately four times that of the rebar, whereas the tensile modulus of elasticity is approximately 0.7 times that of the rebar.

**Figure 3:**
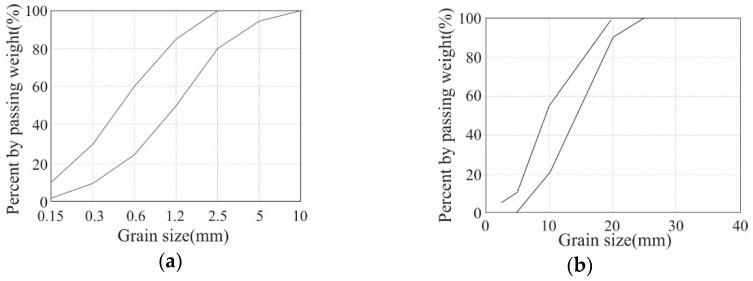
Standard particle size distribution curves: (**a**) fine aggregate; (**b**) coarse aggregate.

Table 2 summarizes the mix design and 28-day compressive strength of the concrete. The designed compressive strength of the concrete is 40 MPa, *w*/*c* ratio is 33%, and the coarse and fine aggregates used follow the standard particle size distribution presented in KS F 2527 [53], as illustrated in Figure 3. The maximum size of the coarse aggregate is 25 mm. The compression test results based on ISO 1920-4 [54] exhibited that the 28-day average compressive strength of the concrete was 45.1 MPa, that is, approximately 13% higher than the design compressive strength.

#### 2.1.2. Specimen Overview

**Figure 4:**
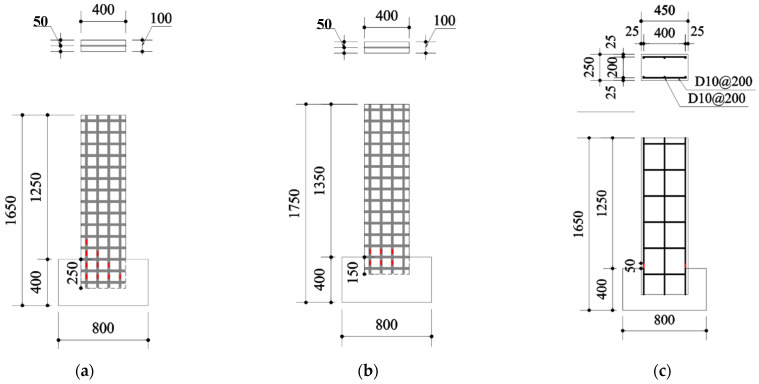
Diagrams of specimens: (**a**) KC_100_F250, KC_100_F250_N06; (**b**) KC_100_F150; (**c**) RC_F250. 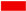 Strain gauges.

Concrete wall specimens reinforced with carbon grid KC (Table 3 and Figure 4) were fabricated to evaluate the applicability of carbon grid KC for concrete structures. The main experimental variables were the ratio of the anchorage length in the foundation to the wall length (0.1, 0.2), and axial force ratio (0.03, 0.06). In addition, an RC wall specimen was fabricated for comparison with the rebar, which is commonly used for concrete reinforcement. The carbon-grid-reinforced concrete specimens were designed with a single layer of carbon grid KC placed at the center of the specimen cross section. Strain gauges were attached to the surfaces of both the CFRP strands and rebars to measure the strain in the CFRP strands of the carbon-grid-reinforced concrete specimens and rebars of the RC specimen (Figure 4). Specifically, the carbon-grid-reinforced concrete specimen with a foundation anchorage length to specimen height ratio of 0.2 had a total of 12 strain gauges attached: 8 at the foundation anchorage location and 4 at the bottom of the wall. The carbon-grid-reinforced concrete specimen with a foundation anchorage length to specimen height ratio of 0.1 had a total of 7 strain gauges attached: 4 at the foundation anchorage location and 3 at the bottom of the wall. Additionally, two strain gauges were attached to the lower part of the wall of the RC specimen.

**Figure 5:**
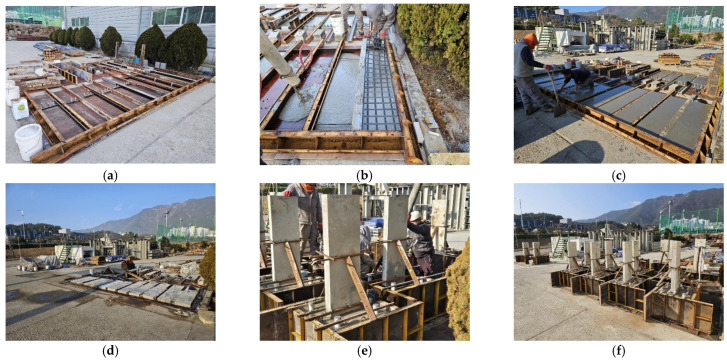
Preparation process for specimens reinforced with carbon grids: (**a**) arrangement of form work for wall construction; (**b**) concrete pouring and arrangement of carbon grid for wall construction; (**c**) completion of concrete pouring for wall construction; (**d**) removal of wall form work; (**e**) erection of wall and concrete pouring for foundation construction; (**f**) completion of concrete pouring for foundation construction.

The carbon-grid-reinforced concrete specimen was fabricated by dividing it into wall and foundation stub sections, as illustrated in Figure 5. First, the wall of the specimen was fabricated by pouring concrete up to the thickness of the mid-section of the cross section, then placing the carbon grid, and finally pouring additional concrete to the full thickness of the specimen cross section. The formwork was removed one day after the concrete was poured. Here, the carbon grid that settles at the foundation was not poured with concrete so that the bottom of the wall would remain exposed. The foundation stub of the specimen was constructed by placing the foundation rebar and erecting the wall above the foundation stub so that the carbon grid exposed at the bottom of the wall was embedded within the foundation (Figure 5e), followed by concrete pouring. The formwork for the base portion was removed one day after concrete placement and cured in the external environment for over 28 days (Figure 5f), until the start of the experiment.

Table 4 summarizes the crack strength and flexural strength of the specimens, which were calculated under the assumption that plane sections remain plane, based on the material test results in Table 1 and Table 2.

Among the carbon-grid-reinforced concrete specimens, the specimens KC_100_F250 and KC_100_F150 with axial force ratios of 0.03 and 0.04, respectively, were expected to fail as a result of the rupture of the carbon grid, whereas the specimen KC_100_F250_N06, with an axial force ratio of 0.06, was expected to fail as a result of the crushing of the cover concrete at the compression edge before the rupture of the carbon grid. RC_F250 was reinforced with rebar to have a flexural strength as similar as possible to that of the carbon-grid-reinforced concrete specimen KC_100_F250 to allow a fair comparison of their flexural behaviors. Final failure as a result of rebar yielding was expected.

In addition, as summarizes in Table 3, the carbon-grid-reinforced concrete specimens had approximately 53% of the reinforcement ratio of the RC specimens, but their flexural strength was 74–86% that of the RC specimens, indicating that the flexural strength was high relative to the reinforcement ratio.

### 2.2. Loading and Measurement Method

**Figure 6:**
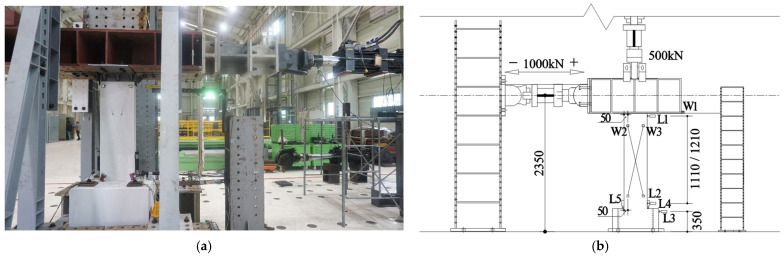
Test setup: (**a**) photo of the setup; (**b**) schematic of loading and measurements.

**Figure 7:**
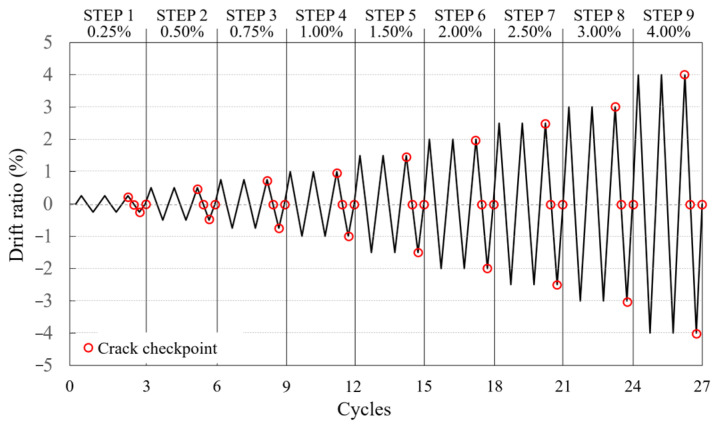
Loading history.

For specimen loading, vertical and horizontal actuators with a capacity of 1000 kN each were used (Figure 6a). After applying a fixed axial force to the top of the specimen using a vertical actuator, positive and negative cyclic loading was performed using a horizontal actuator with displacement control based on the loading history illustrated in Figure 7, which depicts the story drift ratio at the center position of the actuator. Each step was repeated three times with the same story drift ratio.

The vertical and horizontal loads on the specimen were measured using load cells integrated into the actuator. The deformation of the specimen was measured by installing 5 linear variable displacement transducers (LVDTs) (L1–L5) (Tokyo Measuring Instruments Lab, Tokyo, Japan) and 3 wire displacement sensors (W1–W3) (Tokyo Measuring Instruments Lab, Tokyo, Japan), as illustrated in Figure 6b. LVDTs L1–L3 measured the lateral displacements at the top and bottom of the wall and top of the foundation; LVDTs L4 and L5 measured the displacement due to rotation at the bottom of the specimen wall; the wire displacement sensor W1 measured the lateral displacement at the top loading point of the wall; and wire displacement sensors W2 and W3 measured the shear deformation of the wall.

The strain occurring in the CFRP strands of the carbon grid can be measured using strain gauges [55]. Therefore, strain gauges were attached to the surfaces of the longitudinal CFRP strands and rebar to measure the strain. Specifically, 7 to 12 strain gauges were attached to the longitudinal CFRP strands in the foundation and lower part of the walls of the carbon-grid-reinforced concrete specimens (Figure 4a,b). Additionally, the RC specimen had two strain gauges attached to the main reinforcement at the bottom of the wall (Figure 4c).

To evaluate the damage caused to the concrete surface of the specimen by the load, the crack widths at the CFRP strand and main reinforcement locations on the concrete surface were measured using a crack scale (Sinwa Sokutei) at the positive and negative peak points and unloading point (where the load was “0”) during the third cycle of each step (Figure 7).

## 3. Results and Discussion

### 3.1. Crack and Failure Geometry

#### 3.1.1. Failure Geometry

**Figure 8:**
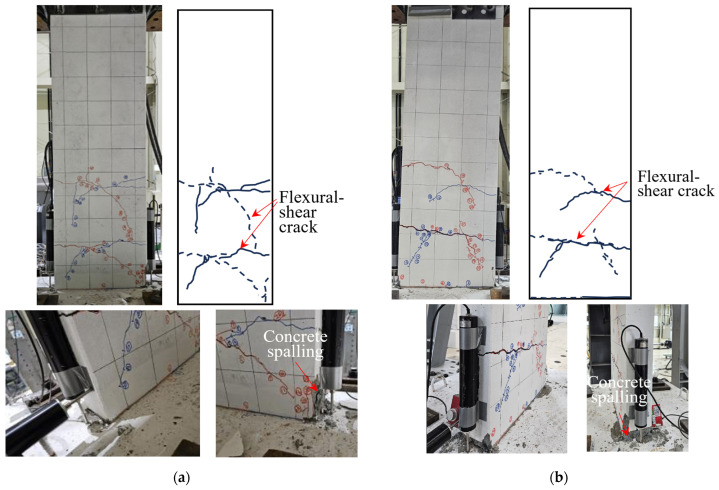

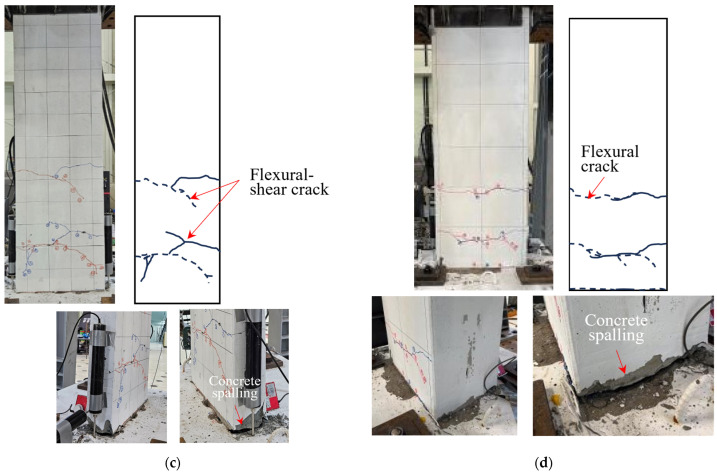
Failure of specimens: (**a**) KC_100_F250; (**b**) KC_100_F250_N06; (**c**) KC_100_F150; (**d**) RC_F250. Red lines and dashed lines represent cracks developed during negative loading, and blue lines and solid lines represent cracks developed during positive loading.

Figure 8 illustrates the damage state of the concrete on the specimen surface after the experiment, where cracks and spalling can be observed. The solid and dashed lines represent cracks that occurred during positive and negative loading, respectively.

In all specimens, cracking first developed at the interface between the bottom of the wall and foundation stub. Subsequently, new flexural cracks developed at the tension edge of the cross section and propagated upward along the height of the specimen. In the carbon-grid-reinforced concrete specimens, the flexural cracks gradually progressed into flexural-shear cracks. No new flexural cracks were developed, the cover concrete at the compression edge of the specimen was crushed, and the cover concrete spalled off. In comparison with RC_F250, flexural-shear cracks developed on the surface of the carbon-grid-reinforced concrete specimen, and the cover concrete at the base of the wall and in narrow area of the foundation stub spalled off.

As illustrated in Figure 8a, KC_100_F250 exhibited longer flexural-shear cracks on its surface than those in the other specimens, and the lower surface concrete of the wall specimen spalled off.

The surface of KC_100_F250_N06 exhibited flexural-shear cracks similar to those in KC_100_F250 (Figure 8b), and the cover concrete at the base of the wall specimen and that in the foundation stub spalled off. As illustrated in Figure 8c, KC_100_F150 developed shorter flexural-shear cracks than those in KC_100_F250, and the cover concrete at the base of the wall specimen as well as in the foundation stub spalled off.

By contrast, RC_F250 exhibited flexural cracking on its surface, instead of flexural–shear cracking, while the extent of cover concrete spalling at the base of the wall specimen and in the foundation stub was greater than that observed in the carbon-grid-reinforced concrete specimens (Figure 8d).

**Figure 9:**
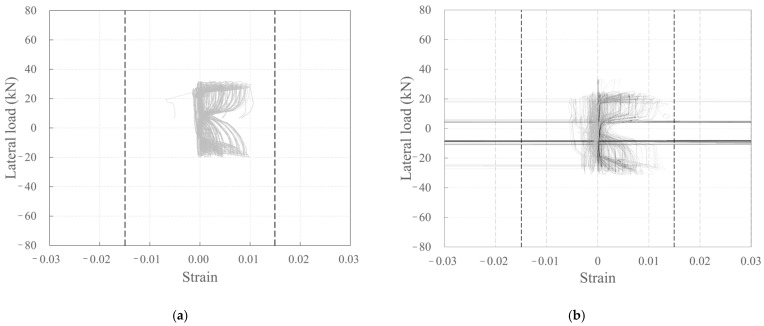

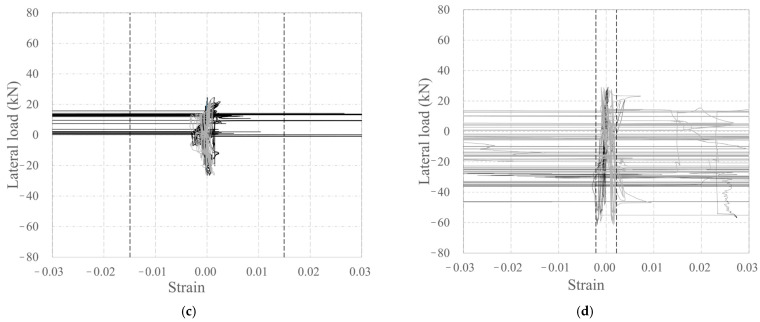
Plots depicting the relationship between lateral load and strain: (**a**) KC_100_F250; (**b**) KC_100_F250_N06; (**c**) KC_100_F150; (**d**) RC_F250.

Figure 9 depicts the relationship between the strain measured by the strain gauges attached to the longitudinal CFRP strands and rebar surfaces of the carbon grid (Figure 3) and the lateral load. Here, the dashed line represents the tensile strain at the tensile strength.

Among the carbon-grid-reinforced concrete specimens, some of the longitudinal CFRP strands of KC_100_F250_N06 and KC_100_F150 experienced tensile strains exceeding those at the tensile strength during the experiment, whereas the longitudinal CFRP strands of KC_100_F250 experienced tensile strains smaller than the tensile strain at the tensile strength until the end of the experiment. Consequently, it is considered that the longitudinal CFRP strands of KC_100_F250_N06 and KC_100_F150 experienced greater stress than that in KC_100_F250. Furthermore, in RC_F250, strain gauges were attached to the rebars at the base of the wall specimen, as illustrated in Figure 4c, and all the rebars with strain gauges attached to them yielded during the experiment.

#### 3.1.2. Crack Formation Characteristics

**Figure 10:**
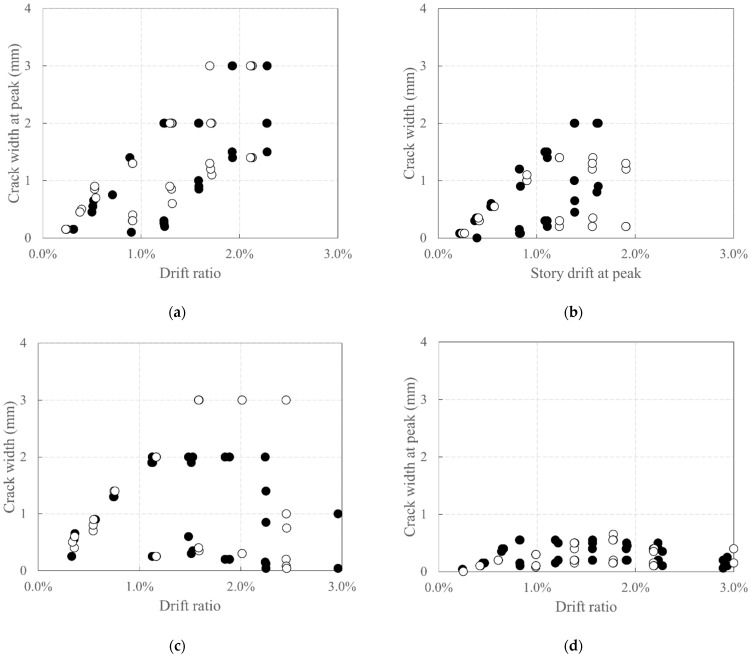
Plots depicting the relationship between crack width and drift ratio at the peak of the cycle: (**a**) KC_100_F250; (**b**) KC_100_F250_N06; (**c**) KC_100_F150; (**d**) RC_F250. White circles represent cracks developed during negative loading, and black circles represent cracks developed during positive loading.

Figure 10 depicts the relationship between the story drift ratio at the peak point of the third cycle at each step and the crack width. Here, the story drift ratio is the difference in lateral displacement measured by the LVDTs L1 and L2 in Figure 6b, divided by the distance between the two LVDTs. In addition, the widths of the cracks that occurred in the wall specimens, excluding those occurring at the interface between the foundation stubs and the base of wall specimens, were measured using a crack scale (Sinwa Sokutei) and were considered as the crack width at the longitudinal CFRP strand and rebar closest to the tension edge.

In RC_F250, the crack width did not increase even after the story drift ratio increased beyond 1.0%. This is believed to be because damage such as crushing and concrete spalling was concentrated in the concrete at the base of the wall specimen and the foundation stub (Figure 8d) resulting in a relatively small increase in the crack width.

In addition, the cracks on the carbon-grid-reinforced concrete specimens exhibited a general tendency to increase with increasing story drift ratio. The crack width on the surface of KC100_F250_N06 was less than 2.0 mm and 1.4 mm under positive and negative loading, respectively. A comparison with the crack width data of the other specimens exhibits that its crack width is smaller at the same story drift ratio. This is believed to be because of the larger axial force acting on this specimen than that on the others, suppressing the widening of the crack width [56]. KC100_F250 exhibited cracks with a width of 3.0 mm or less under both positive and negative loading, whereas KC100_F150 exhibited cracks with widths of 2.0 mm and 3.0 mm or less under positive and negative loading, respectively. This indicates that the difference in crack width owing to the difference in the ratio of the anchorage length in the foundation to the specimen height was minimal.

**Figure 11:**
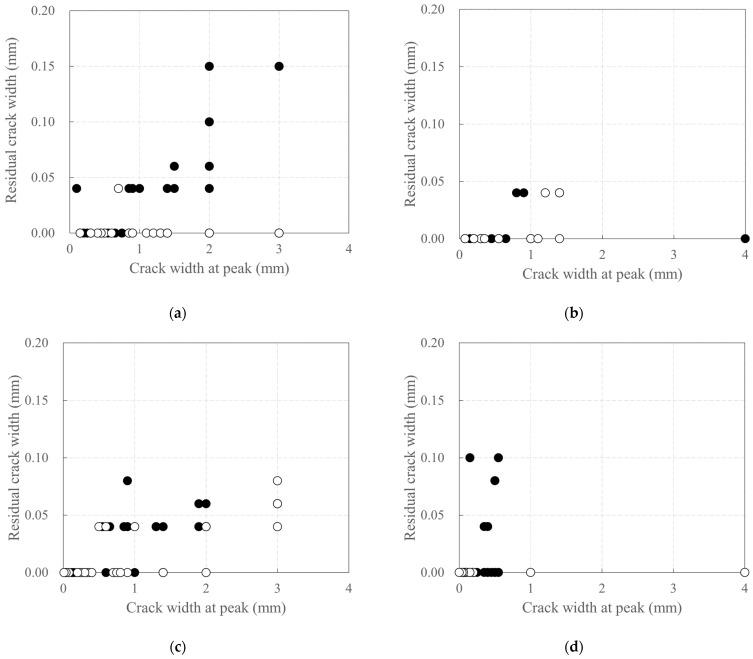
Plots depicting the relationship between residual crack width and crack width at the peak of the cycle: (**a**) KC_100_F250; (**b**) KC_100_F250_N05; (**c**) KC_100_F150; (**d**) RC_F250. White circles represent cracks developed during negative loading, and black circles represent cracks developed during positive loading.

Figure 11 depicts the relationship between the crack width at the peak of the third cycle for each step and the residual crack width at the unloading point. The residual crack width of the carbon-grid-reinforced concrete specimens was found to be smaller than that of specimen RC_F250, except for KC_100_F250. The ratio of the residual crack width to peak crack width for KC_100_F250 was less than 0.400, which is smaller than the corresponding value for specimen RC_F250 (0.667). This indicates that the carbon-grid-reinforced concrete specimen closed a significant portion of the crack at the unloading point, even though the peak crack width was large.

Among the carbon-grid-reinforced concrete specimens, the residual crack width of specimen KC_100_F250_N06, which had the smallest peak crack width, was less than 0.06 mm and 0.04 mm under positive and negative loading, respectively. Even with similar peak crack widths, it exhibited the smallest residual crack width among the carbon-grid-reinforced concrete specimens. Additionally, the ratio of the residual crack width to peak crack width was found to be the smallest (0.036 or less). This is likely because the residual crack width did not increase due to the higher axial load than that of the other carbon-grid-reinforced concrete specimens [56].

Furthermore, the residual crack width of KC_100_F150 was 0.08 mm under both positive and negative loading, and the ratio of the residual crack width to peak crack width was less than 0.089, indicating that the crack closed more effectively than in KC_100_F250.

### 3.2. Hysteresis Properties

#### 3.2.1. Hysteresis Curve

**Figure 12:**
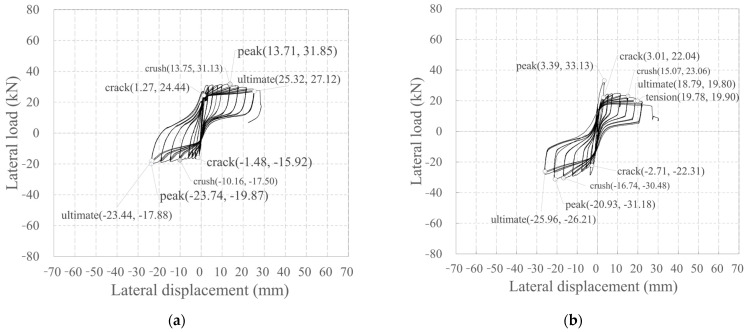

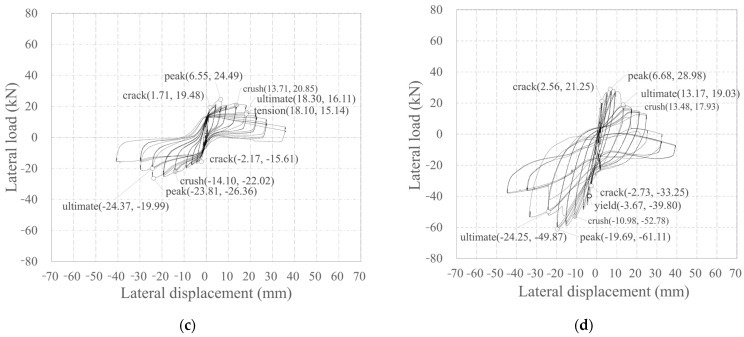
Plots depicting the relationship between lateral load and displacement: (**a**) KC_100_F250; (**b**) KC_100_F250_N06; (**c**) KC_100_F150; (**d**) RC_F250. Crack: point of initial crack formation; crush: point of concrete crushing at the compression edge; peak: point of maximum load; tension: point where the tensile strain of the longitudinal CFRP strands in the carbon grid reaches the tensile strain at the tensile strength; ultimate: point of final failure, when the load has decreased to 80% of the peak load.

Figure 12 depicts the relationship between the lateral load and lateral displacement of the specimen. Here, lateral displacement is the difference between the lateral displacements measured by the LVDTs L1 and L2 (Figure 6b).

KC_100_F250 developed its first crack during the −4 cycle (the first cycle of step 2). It reached its peak load without any significant increase under repeated loading. During positive loading, a crack also developed during the +5 cycle (the second cycle of step 2), which reached its peak load without any significant increase under repeated loading. As the peak load was reached, the cover concrete at the compression edge was crushed, which decreased the load and ultimately resulted in final failure.

KC_100_F250_N06 developed its first crack during the 4th cycle (the first cycle of step 2) in both the positive and negative directions, and the load increased until the 6th cycle (the third cycle of step 2). Subsequently, during positive loading, the load rapidly decreased, then slowly increased until the cover concrete was crushed in the compression edge, after which the load decreased again, leading to final failure. During negative loading, the load rapidly decreased in the −7 cycle (the first cycle of step 3), but then slowly increased, reaching a peak load even after the cover concrete at the compression edge was crushed.

Similarly to KC_100_F250_N06, KC_100_F150 developed its first crack during the +4 cycle (the first cycle of step 2), which reached its peak load in the +7 cycle (the first cycle of step 3). Subsequently, it experienced a rapid decrease in load, after which the load gradually increased during positive loading and then began to decrease due to crushing of the cover concrete at the compression edge. At final failure, the tensile strain of the longitudinal CFRP strands reached the tensile strain at the tensile strength. Even under negative loading, the first crack occurred during the −4 cycle (the first cycle of step 2). Thereafter, the peak load was reached without any significant increase under repeated loading. After reaching the peak load, the specimen failed in the next cycle under the same story drift ratio, with the load decreasing to less than 80% of the peak load. This is believed to be because of the tensile strain of the longitudinal CFRP strands during positive loading before the peak, reaching the tensile strain at the tensile strength.

RC_F250 experienced initial cracking in both positive and negative directions during the 2nd cycle (the second cycle of step 1), which occurred sooner than in the case of the carbon grid. Subsequently, the rebar yielded at a relatively small displacement of −1.67 mm (drift ratio = 0.15%). Moreover, during positive loading, the load decreased rapidly after reaching the peak, and the compression-edge cover concrete crushed simultaneously with final failure. Under negative loading, the load increased and reached its peak load even after the cover concrete at the compression edge had been crushed; as in the case of positive loading, it rapidly decreased after reaching the peak load, resulting in final failure.

Table 5 summarizes the damage and failure process of the specimens under the positive and negative loading in Figure 12. As specified in the specimen design in Table 4, RC_250 reached its peak load and ultimately failed after both the positive and negative rebars yielded.

However, unlike the specimen design in Table 4, all the carbon-grid concrete specimens experienced crushing of the cover concrete at the compression edge before the tensile strain of the longitudinal CFRP strands reached the tensile strain at tensile strength, which led to the appearance of the peak. Furthermore, unlike KC_100_F250, which was predicted to gain flexural strength due to longitudinal CFRP strand failure (Table 4), the tensile strain of the longitudinal CFRP strands did not reach the tensile strain at the tensile strength by the end of the experiment. However, in KC_100_F250_N06, where the axial force was twice that of KC_100_F250, and KC_100_F150, where the ratio of the anchorage length in the foundation to the specimen height was low at 0.1, the tensile strain of the longitudinal CFRP strands reached the tensile strain at tensile strength simultaneously with final failure. After crushing of the compression-edge cover concrete, the carbon grid resists the load. It is inferred that the longitudinal CFRP strands failed under tension at a load level below 80% of the maximum load, particularly with significant damage in the form of spalling of the cover concrete in KC_100_F250_N06 and KC_100_F150 when compared with that in the case of specimen CF100_F250, as illustrated in Figure 8.

#### 3.2.2. Energy Absorption

**Figure 13:**
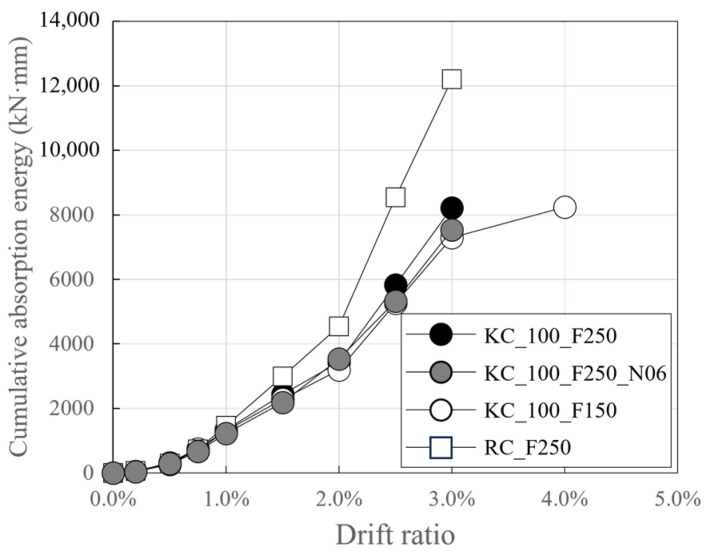
Cumulative absorption energy of specimens.

Figure 13 depicts the change in cumulative absorbed energy of the specimen due to loading, based on the target story drift ratio for each step. Here, the absorbed energy per step is the sum of the energy absorbed during the 3-cycle loading under the same target story drift ratio. The absorbed energy of the specimens was calculated by multiplying the displacement and load derived from the hysteresis curves in Figure 12.

For story drift ratios below the target of 1.0%, the difference in cumulative absorbed energy between the specimens at the same target story drift ratio was found to be negligible. However, in the range where the target story drift ratio was greater than or equal to 1.0%, the cumulative absorbed energy of RC_250 began to exceed that of the carbon-grid-reinforced concrete specimen. The energy absorbed by the RC specimen up to the target story drift ratio of 3.0% at the final failure point was 1.47 to 1.67 times the energy absorbed by the carbon-grid-reinforced specimen. It is believed that the energy absorption increased as the load on RC_250 increased at similar displacement levels, as well as severe damage, particularly cover concrete spalling at the base of the wall specimen and in the foundation stub (Figure 8d).

In addition, among the carbon-grid-reinforced concrete specimens, CF100_F250 exhibited the largest final cumulative absorbed energy of 8220 kNmm; this value was 1.09 times and 1.13 times larger than that of CF100_F250_N06, which had twice the axial force, and CF100_F150, which had a low ratio of the anchorage length in the foundation to the specimen height of 0.1, respectively. As illustrated in Figure 8a–c and Figure 10a–c, CF100_F250 exhibited greater damage, such as cracks and cover concrete spalling, on its surface when compared with the other carbon-grid-reinforced concrete specimens, which is believed to have resulted in a larger cumulative absorbed energy.

#### 3.2.3. Equivalent Stiffness

**Figure 14:**
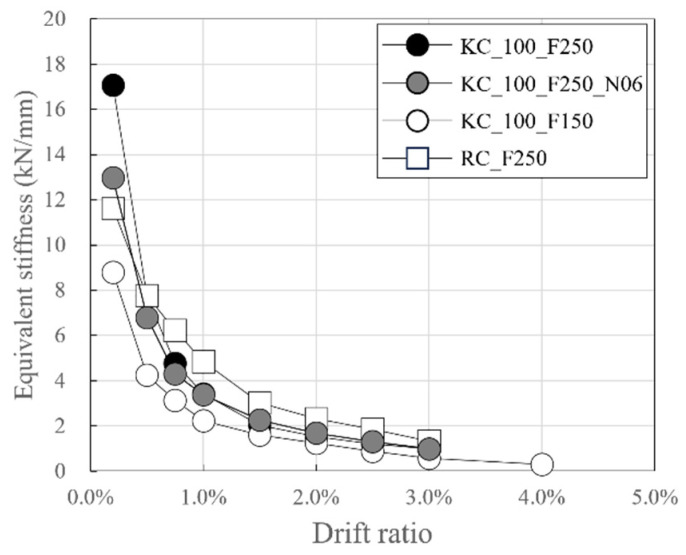
Variation in equivalent stiffness of the specimens.

Figure 14 depicts the change in equivalent stiffness as a function of the drift ratio. Here, the equivalent stiffness is the slope of the straight line connecting the positive and negative peak points of the first cycle for each target drift ratio.

RC_F250 had the largest cross-sectional height and width among the specimens, but its initial stiffness was found to be smaller than those of the carbon-grid-reinforced concrete specimens KC_100_F250 and KC_100_F250_N06. However, during the loading in step 2, all carbon-grid-reinforced concrete specimens exhibited initial cracks, leading to a significant decrease in their stiffness. Consequently, the equivalent stiffness also decreased significantly, and by the end of the experiment, the equivalent stiffness of the carbon-grid-reinforced concrete specimens was found to be smaller than that of RC_F250.

At the ratio of the anchorage length in the foundation to wall height of 0.2, the equivalent stiffnesses after step 2 for KC_100_F250 and KC_100_F250_N06 were found to be similar. KC_100_F150 had the smallest equivalent stiffness, ranging from 0.51 to 0.82 of that of KC_100_F250 at each step, with an anchorage length in the foundation to wall height ratio of 0.1. According to previous research, it is known that if the anchorage length in the foundation is small, the rebar slip increases, making it difficult to maintain the stiffness [57]. Similarly, it is believed that as the anchorage length in the foundation of the carbon grid shortens, the carbon-grid slip increases, leading to a decrease in stiffness.

### 3.3. Skeleton Curve

Table 6 summarizes the lateral load, lateral displacement, and story drift ratio at the crack, crush, peak, tension, and ultimate points in Figure 12 for each specimen.

The maximum load of KC_100_F250_N06 was 1.04 times larger than that of KC_100_F250 during positive loading, 1.57 times larger during negative loading, and 1.30 times larger on average. Further, in the case of positive loading of KC_100_F250_N06, the load rapidly decreased at the peak of the +7 cycle (the first cycle of step 3) after reaching the maximum load during the +6 cycle (the third cycle of step 2), then gradually increased until the cover concrete at the compression edge was crushed. The load at the crushing of the compression-edge cover concrete was 0.74 times the positive maximum load of KC_100_F250; although small, it was 1.74 times larger than the negative maximum load and, on average, 1.24 times larger than the average maximum loads in both positive and negative loading of KC_100_F250. This is known to occur in the RC members, wherein the neutral axis lengthens and cross-sectional area under compression increases when axial force is applied, leading to an increase in flexural strength up to an appropriate level [58]. Similarly, it is believed that the flexural strength of KC_100_F250_N06 increased due to the application of larger axial force.

The maximum load of KC_100_F150 was found to be 0.77 times smaller than that of KC_100_F250 during positive loading and 1.33 times larger during negative loading. However, the average maximum load was found to be similar at 1.02 times, indicating that the difference in maximum load due to the difference in anchorage length in the foundation was minimal.

**Figure 15:**
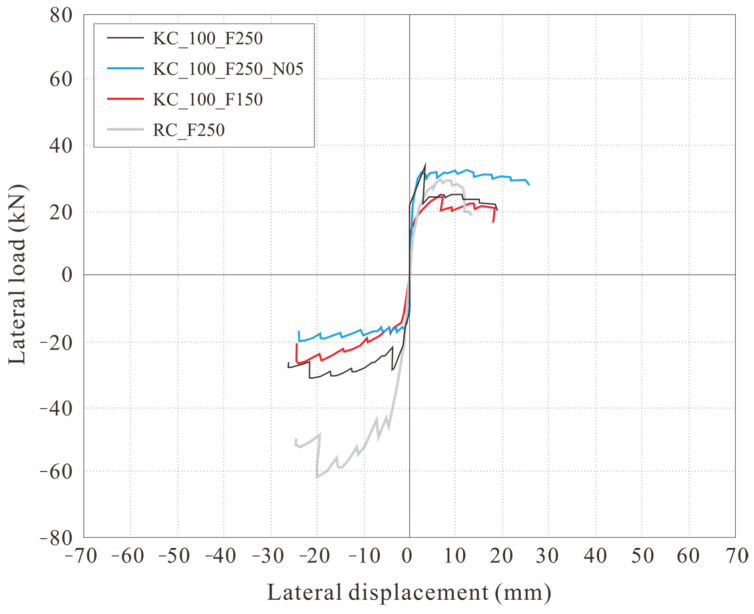
Skeleton curves of the specimens.

Figure 15 depicts the skeleton curves for each specimen. The skeleton curve for KC_100_F250 exhibited an increase in load during positive loading, whereas those of KC_100_F250_N06 and KC_100_F150 exhibited an increase in load during negative loading.

In comparison with RC_F250, the carbon-grid-reinforced concrete specimens exhibited a significant decrease in stiffness due to cracking, as shown in Figure 15, and reached their maximum load. However, the deformation until final failure was 1.39–1.92 times greater under positive loading and 0.97–1.07 times greater under negative loading when compared with the data for RC_F250, indicating that the carbon-grid-reinforced concrete specimens had greater deformation capacity than that of the RC specimen.

**Figure 16:**
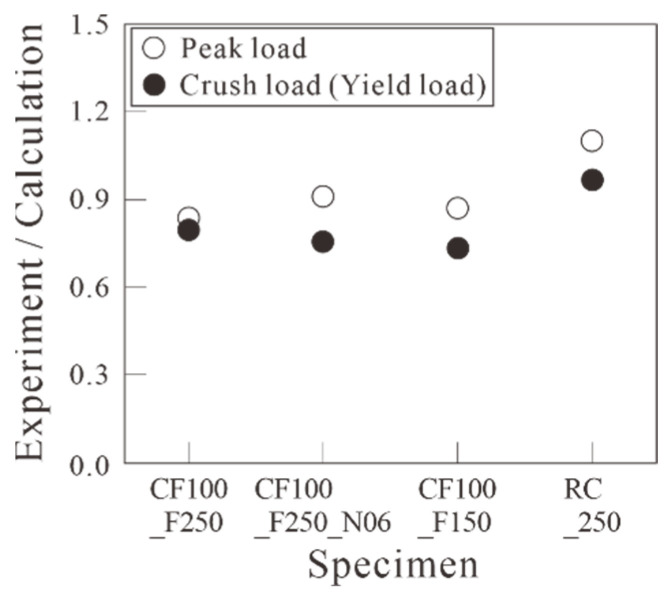
Load ratio of experimental and calculated results for specimens.

As depicted in Figure 16, the average values of the positive and negative maximum loads and the load at rebar yield for RC_F250 are 1.09 and 0.96 times the calculated flexural strength values, respectively, based on the assumption of the plane section remains plane in Table 4. This indicates that the maximum load was higher than the calculated value, but the load at yield was lower. However, in the carbon-grid-reinforced concrete specimens, the average values of the positive and negative maximum loads and the load at crush were 0.85–0.91 and 0.73–0.80 times the calculated flexural strength values, respectively, based on the assumption of plane section remains plane in Table 4. This indicates that the flexural strength obtained from the experimental results was lower than the calculated values.

**Figure 17:**
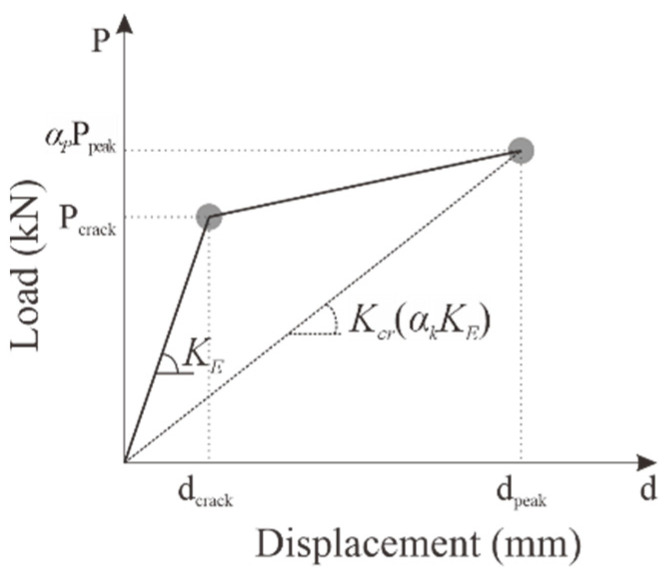
Idealized bilinear skeleton curve. *K_E_*: initial stiffness before crack initiation; *K_cr_*: cracking stiffness, defined as the slope of the line connecting the peak point to the origin; *α_k_*: ratio of *K_cr_* to *K_E_*; *P_crack_* and *d_crack_*: load and displacement at crack initiation, respectively; *P_peak_* and *d_peak_*: maximum load due to concrete crushing at the compression edge and its corresponding displacement, respectively; *α_p_*: strength reduction factor.

As presented in Figure 12 and Table 5, KC100_F250_N06 reached its maximum load at a relatively small deformation during positive loading, after which the load rapidly decreased and then gradually increased until the cover concrete at the compression edge was crushed. However, the carbon grid-reinforced concrete specimens exhibited a significant decrease in stiffness as the cracks occurred, reaching their peak load when the cover concrete at the compression edge was crushed. Therefore, the skeleton curve of the carbon-grid-reinforced concrete specimen is considered to be defined as a bilinear line, as illustrated in Figure 17, where the stiffness decreases because of cracking and the maximum load is reached because of crushing.

The experimental results indicated that positive and negative compression-edge cover concrete crushing occurred in the same cycle for all carbon-grid-reinforced concrete specimens. Therefore, *K_cr_* can be defined as the equivalent stiffness at the cycle where compressive-edge concrete crushing occurred. Consequently, the *α_k_* values for each specimen were found to be in the range of 0.12 to 0.18 (Table 7). KC_100_F150 exhibited the highest stiffness after crack initiation among the carbon-grid-reinforced concrete specimens, as depicted in Figure 15, resulting in the highest ***α_k_*** value.

The strength reduction factor, *α_p_*, is defined as the ratio of the load at the crush point—calculated as the average of the positive and negative loads (Table 6)—to the calculated flexural strength value (Table 4), which is based on the assumption of plane sections remain plane. The *α_p_* values for each specimen ranged from 0.73 to 0.80 (Table 7). Although the strength reduction factor for KC_100_F150, where the ratio of the anchorage length in the foundation to wall height was 0.1, was the smallest, a notable difference in the values was not observed between the specimens.

For concrete members, the initial stiffness *K_E_* in the lateral load–displacement relationship can be obtained using the following Equation (1):

(1) $$K_{E}=\frac{12E_{c}I_{g}}{L^{3}},$$

where *E_c_*: modulus of elasticity of concrete (MPa), *I_g_*: gross moment of inertia (mm^4^), *L*: specimen span length (mm).

**Figure 18:**
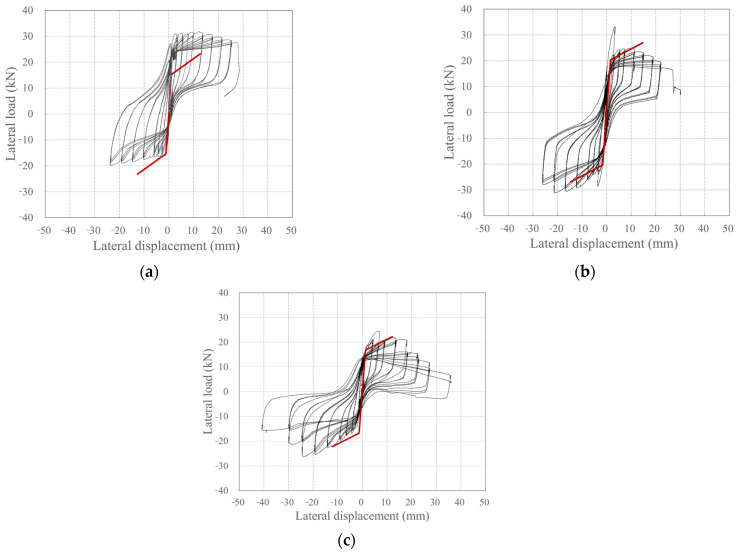
Comparison of skeleton curves of specimens: (**a**) KC_100_F250; (**b**) KC_100_F250_N06; (**c**) KC_100_F150.

Figure 18 compares the calculated crack strength and flexural strength from Table 4, skeleton curve estimated using the average values of *K_E_*, *α_p_*, and *α_k_* from Equation (1), and lateral load–displacement relationship obtained from the experimental results.

In the case of KC100_F250, the skeleton curves differed between positive and negative loading because a larger load occurred at the same deformation during positive loading than that in negative loading. Consequently, the bilinear skeleton curve in Figure 17 is smaller than the experimental results during positive loading and larger than the experimental results during negative loading. However, the bilinear skeleton curves of KC100_F250_N06 and KC100_F150 in Figure 17 broadly simulate the skeleton curve obtained from the experimental results until the crush point is reached.

### 3.4. Deformation Properties

The overall deformation of the specimen can be considered as being divided into flexural deformation at the bottom of the wall specimen and shear deformation in the central part [59].

The flexural deformation at the bottom of the wall specimen is the rotation angle caused by the moment generated by the lateral load. The rotation angle at the bottom of the wall specimen is the difference in displacement measured by the LVDTs L4 and L5 in Figure 5b divided by the distance between them.

**Figure 19:**
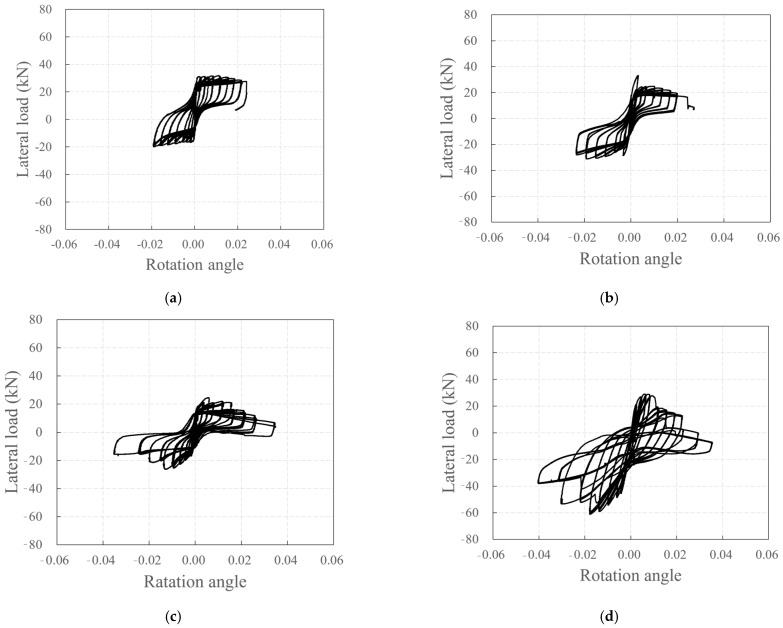
Plots depicting the relationship between lateral load and rotation angle: (**a**) KC_100_F250; (**b**) KC_100_F250_N06; (**c**) KC_100_F150; (**d**) RC_F250.

The relationship between the lateral load and rotation angle at the base of all wall specimens, depicted in Figure 19, resembles the lateral load–displacement relationship depicted in Figure 12. This suggests that significant lateral displacements occurred in all specimens due to the rotation at the bottom of the wall.

In addition, the shear deformation in the central part of the specimen can be obtained using the displacement data measured by the wire displacement sensors W2 and W3 (Figure 5b) and the following Equation (2):

(2) $$\gamma =\frac{1}{2}(\frac{\sqrt{h^{2}+l^{2}}}{l})(\frac{\Delta_{1}}{h_{1}}-\frac{\Delta_{2}}{h_{2}})$$

where Δ_1_, Δ_2_ Change in diagonal length of the shear measurement (measurements of wire displacement sensors W2 and W3 in Figure 5b); *h*: Vertical distance of shear measurement (vertical distance of wire displacement sensors W2 and W3 in Figure 5b); *l*: Horizontal distance of shear measurement (horizontal distance of wire displacement sensors W2 and W3 in Figure 5b).

**Figure 20:**
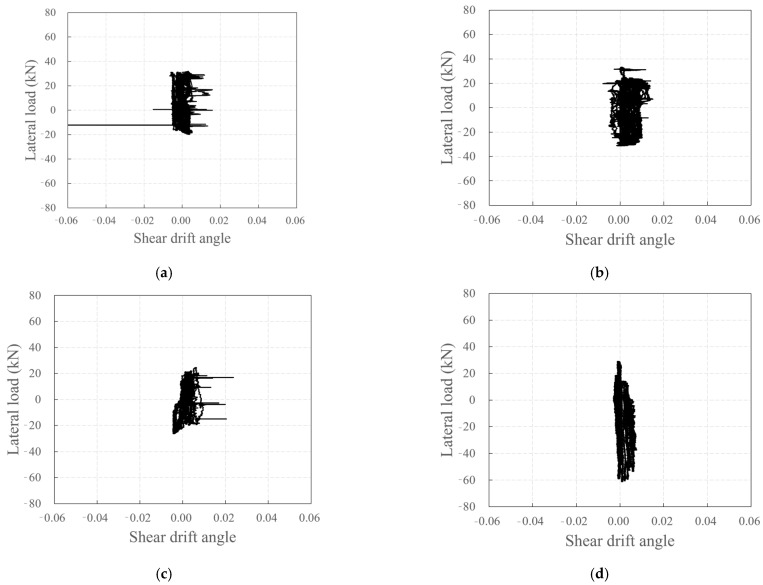
Plots depicting the relationship between lateral load and shear drift angle: (**a**) KC_100_F250; (**b**) KC_100_F250_N06; (**c**) KC_100_F150; (**d**) RC_F250.

Figure 20 depicts the relationship between the lateral load and shear drift angle according to Equation (2). The shear drift angle did not increase significantly with increase in the lateral load, in contrast to the rotation angle at the bottom of the wall specimen. The carbon-grid-reinforced concrete specimen, which exhibited flexural-shear cracks, showed a larger shear drift angle than that in RC_F250, which only exhibited flexural cracks. The noise in Figure 20a–c is also considered to be due to the occurrence of flexural-shear cracks within the shear strain measurement section.

**Figure 21:**
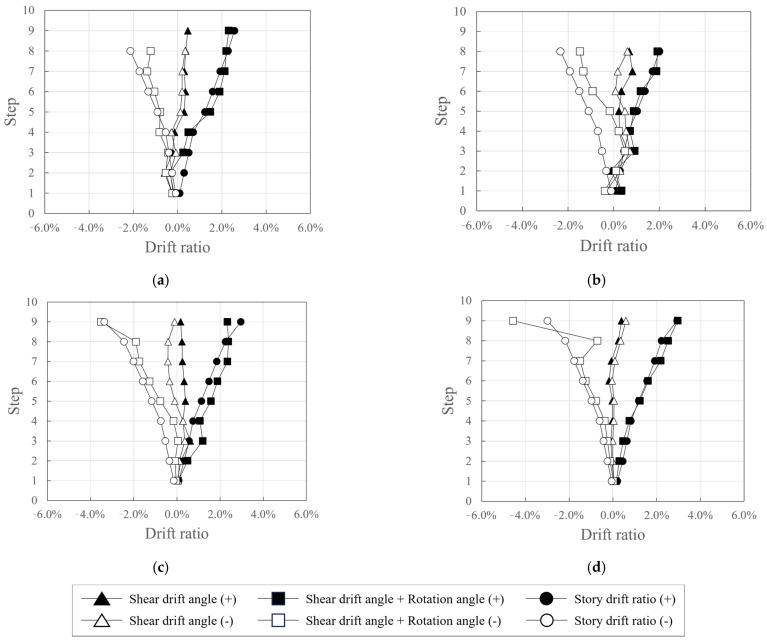
Comparison of the proportions of shear drift angle, rotation angle, and drift ratio: (**a**) KC_100_F250; (**b**) KC_100_F250_N06; (**c**) KC_100_F150; (**d**) RC_F250.

Figure 21 shows the shear drift angles at the peak points for each step and the sum of the shear drift angle and rotation angle at the bottom of the wall specimen as functions of the story drift ratio.

Although there were some differences, the shear drift angle was smaller than the bottom rotation angle of the wall specimens in both positive and negative directions in all specimens. The bottom rotation angle of the wall specimens accounted for most of the story drift ratio, and the sum of the shear drift angle and bottom rotation angle of the wall specimens was similar to the story drift ratio. Furthermore, the difference between the sum of the shear drift angle and rotation angle at the bottom of the wall specimen and the story drift ratio is considered to be due to the widening of the crack width.

The rotation angle at the bottom of the wall specimen for KC_100_F250_N06 was small in the relatively low-deformation sections where the story drift ratio was less than 1% during negative loading. However, in sections where the story drift ratio was 1% or higher, the rotation angle at the bottom of the wall specimen generally accounted for a significant portion of the total deformation, and the sum of the shear drift angle and bottom rotation angle was found to be similar to the story drift ratio.

## 4. Conclusions

This study evaluated the seismic behavior of carbon grid-concrete wall specimens through quasi-static cyclic tests by using carbon grid KC as the reinforcement for the concrete walls. The experimental variables were the ratio of the anchorage length in the foundation to the wall length and axial force ratio. The results are as follows:

1. Unlike the RC specimen, the carbon-grid-reinforced concrete specimens experienced crack progression from flexural cracks to flexural-shear cracks, and the area of spalling concrete at the bottom of the wall and foundation stub was smaller. Among the carbon-grid-reinforced concrete specimens, those with higher axial loads exhibited less surface damage, such as crack length, width, and spalling concrete. Additionally, the crack length decreased as the ratio of the anchorage length of the carbon grid in the foundation to the wall length decreased.
2. When the axial force was large, the maximum crack width was 2 mm, indicating that the axial force suppressed the widening of the crack width. In addition, the ratio of the residual crack width to the peak crack width was 0.400 or less, which is lower than that of the RC specimen (0.667). Therefore, the cracks that formed on the surface of the carbon-grid-reinforced concrete specimen closed by a greater extent than that in the RC specimen.
3. In the carbon-grid-reinforced concrete specimens, the stiffness significantly decreased due to cracking, and the maximum load was generally reached due to the compression-edge cover concrete crushing with the average ratio of the equivalent stiffness at the crushing of the compression-edge cover concrete to the initial stiffness, 0.14.
4. The load at the crushing of the compression-edge cover concrete of the carbon-grid-reinforced concrete specimen was approximately 0.73 to 0.80 times the flexural strength calculated based on the assumption of plane sections remaining plane.
5. The cumulative absorbed energy of the carbon-grid-reinforced concrete specimens with a target story drift ratio of 3.0% up to failure was found to be 0.60–0.62 times the cumulative absorbed energy of the RC specimen.
6. The skeleton curve of the carbon-grid-reinforced concrete specimen can be defined as bilinear, with the stiffness decreasing due to crack initiation, and the crushing of the compression-edge cover concrete resulting in a peak. The skeleton curve estimated using the ratio of the cracking stiffness to the initial stiffness and average value of the strength reduction factor, obtained from the experimental results, exhibited differences between positive and negative loading but generally enveloped the skeleton curves obtained in the experiments of this paper.
7. The carbon-grid-reinforced concrete specimens exhibited a lower maximum load and cumulative absorbed energy than those of the RC specimen, but the damage area was smaller, and the deformation capacity was greater until the end of the experiment. Therefore, the carbon grid KC is considered suitable for use as reinforcement for concrete. However, the results are based on limited experimental data (reinforcing ratio of 0.20, axial force ratio of 0.03 and 0.06, and the ratio of the carbon-grid anchorage length in foundation to wall length of 0.2 and 0.1). Further experimental and analytical studies, along with a comprehensive analysis of the existing data, are required for the future seismic design of carbon-grid-reinforced members.

## Figures and Tables

**Table 1:** Characteristics and tensile properties of reinforcements.

Name	Strand			Grid Geometry (Longitudinal (G__VS_) × Transverse (G__TS_) Spacing, mm)	Tensile Strength (MPa)	Tensile Modulus of Elasticity (MPa)	Tensile Strain
	Width (mm)	Thickness (mm)	Area (mm^2^)				
KC	20	1	20	100 × 100	2271	151,904	0.013
D10	-	-	71.3	-	582 (469 *)	216,657	-

* Yield strength.

**Table 2:** Mix design and compressive strengths of concrete.

Cement (kg/m^3^)	Water (kg/m^3^)	W/C (%)	Fine Aggregate (kg/m^3^)	Coarse Aggregate (kg/m^3^)	Superplasticizer (kg/m^3^)	Compressive Strength (MPa)
500	165	33	760	934	5	45.1

**Table 3:** Specifications of wall specimens.

Specimen	Length (mm)	Width (mm)	Thickness (mm)	Anchorage Length in Foundation (mm)	Anchorage Length in Foundation/Wall Length	Reinforcement	Reinforcement Ratio (%)	Axial Force Ratio (Axial Force, kN)
KC_100_F250	1250	400	100	250	0.2	KC	0.20	0.03 (59.4)
KC_100_F250_N06	1250							0.06 (106.4)
KC_100_F150	1350			150	0.1			0.04 (73.1)
RC_F250	1250		250	250	0.2	d10@200 (longitudinal direction) × d10@200 (transverse direction	0.38	0.02 (81.0)

**Table 4:** Crack and flexural strength calculation results of the specimens.

Specimen	Crack Strength		Flexural Strength		Failure Mode
	Moment (kNm)	Lateral Load (kN)	Moment (kNm)	Lateral Load (kN)	
KC_100_F250	11.3	9.0	38.1	30.5	CFRP strand rupture
KC_100_F250_N06	11.3	9.0	44.3	35.4	Concrete crushing
KC_100_F150	11.3	8.4	39.5	29.3	CFRP strand rupture
RC_F250	35.7	23.8	51.6	41.3	Rebar yielding

**Table 5:** Failure process of the specimens.

Specimen	Loading Direction	Failure Mode
KC_100_F250	Positive	Crack → Crush, Peak → Ultimate
	Negative	Crack → Crush, Peak → Ultimate
	(Final)	Concrete crushing
KC_100_F250_N06	Positive	Crack → Peak → Crush → Ultimate, Tension
	Negative	Crack → Crush → Peak → Ultimate
	(Final)	CFRP strand rupture after concrete crushing
KC_100_F150	Positive	Crack → Peak → Crush → Ultimate, Tension
	Negative	Crack → Crush → Peak → Ultimate
	(Final)	CFRP strand rupture after concrete crushing
RC_250	Positive	Crack → Yield → Peak → Ultimate, Crush
	Negative	Crack → Yield → Crush → Peak → Ultimate
	(Final)	Rebar yielding

**Table 6:** Load and story drift results.

Loading Direction	Specimen	Crack			Crush			Peak			Tension			Ultimate		
		*P* (kN)	*D* (mm)	*R*	*P* (kN)	*D* (mm)	*R*	*P* (kN)	*D* (mm)	*R*	*P* (kN)	*D* (mm)	*R*	*P* (kN)	*D* (mm)	*R*
Positive	CF100_F250	24.4	1.27	0.0011	31.1	13.75	0.0124	31.9	13.71	0.0124	-	-	-	27.1	25.32	0.0228
	CF100_F250 _N06	22.1	3.01	0.0022	23.1	15.07	0.0136	33.1	3.39	0.0031	19.9	19.78	0.0178	19.8	18.79	0.0169
	CF100_F150	19.5	1.71	0.0014	20.8	13.71	0.0113	24.5	6.55	0.0054	15.1	18.10	0.0150	16.1	18.30	0.0151
	RC_250	21.3	2.56	0.0023	17.9	13.48	0.0121	29.0	6.68	0.0060	-	-	-	19.0	13.17	0.0119
Negative	CF100_F250	−15.9	−1.48	−0.0013	−17.5	−10.16	−0.0091	−19.9	−23.74	−0.0214	-	-	-	−17.9	−23.44	0.0228
	CF100_F250 _N06	−22.7	−2.71	−0.0027	−30.5	−16.74	−0.0151	−31.2	−20.93	−0.0189	-	-	-	−26.2	−25.96	−0.0234
	CF100_F150	−15.6	−2.17	−0.0018	−22.0	−14.10	−0.0117	−26.4	−23.81	−0.0197	-	-	-	−20.0	−24.37	−0.0201
	RC_250	−33.3	−2.73	−0.0025	−52.8	−10.98	−0.0099	−61.1	−19.69	−0.0177	−39.8	−3.67	−0.0033	−49.9	−24.25	−0.0218

*P*: Lateral load, *D*: Lateral displacement, *R*: Drift ratio.

**Table 7:** Characteristic values of the skeleton curve.

Specimen	*α_p_*	Stiffness (kN/mm)		*α_k_*
		*K_E_*	*K_cr_*	
KC_100_F250	0.80	17.07	2.03	0.12
KC_100_F250_N05	0.76	12.98	1.68	0.13
KC_100_F150	0.73	8.79	1.60	0.18
average	0.76			0.14

## Data Availability

The original contributions presented in this study are included in the article. Further inquiries can be directed to the corresponding authors.

## Abbreviations

The following abbreviations are used in this manuscript:

CFRP	Carbon fiber-reinforced polymer
FRP	Fiber-reinforced polymer
LVDT	Linear Variable Displacement Transducer
MMA	Methyl methacrylate
RC	Reinforced concrete
TRC	Textile-reinforced concrete
